# Brassinosteroids play a critical role in the regulation of pesticide metabolism in crop plants

**DOI:** 10.1038/srep09018

**Published:** 2015-03-12

**Authors:** Yanhong Zhou, Xiaojian Xia, Gaobo Yu, Jitao Wang, Jingxue Wu, Mengmeng Wang, Youxin Yang, Kai Shi, Yunlong Yu, Zhixiang Chen, Jay Gan, Jingquan Yu

**Affiliations:** 1 Department of Horticulture, Zijingang Campus, Zhejiang University, 866 Yuhangtang Road, Hangzhou, 310058, P.R. China; 2 Institute of Pesticide & Environmental Toxicology, Zijingang Campus, Zhejiang University, 866 Yuhangtang Road, Hangzhou, 310058, P.R. China; 3 Department of Botany & Plant Pathology, Purdue University, West Lafayette, IN 47907-2054, USA; 4 Department of Environmental Science, University of California Riverside, Riverside, CA 92521, USA

## Abstract

Pesticide residues in agricultural produce pose a threat to human health worldwide. Although the detoxification mechanisms for xenobiotics have been extensively studied in mammalian cells, information about the regulation network in plants remains elusive. Here we show that brassinosteroids (BRs), a class of natural plant hormones, decreased residues of common organophosphorus, organochlorine and carbamate pesticides by 30–70% on tomato, rice, tea, broccoli, cucumber, strawberry, and other plants when treated externally. Genome-wide microarray analysis showed that fungicide chlorothalonil (CHT) and BR co-upregulated 301 genes, including a set of detoxifying genes encoding cytochrome P450, oxidoreductase, hydrolase and transferase in tomato plants. The level of BRs was closely related to the *respiratory burst oxidase 1* (*RBOH1*)-encoded NADPH oxides-dependent H_2_O_2_ production, glutathione biosynthesis and the redox homeostasis, and the activity of glutathione *S*-transferase (GST). Gene silencing treatments showed that BRs decreased pesticide residues in plants likely by promoting their metabolism through a signaling pathway involving BRs-induced H_2_O_2_ production and cellular redox change. Our study provided a novel approach for minimizing pesticide residues in crops by exploiting plants' own detoxification mechanisms.

Pesticides are important chemicals used in modern agriculture for protection against pests and weeds, as up to 80% of crop yields could be lost without pesticides^1^. The extensive use of pesticides has resulted in contamination of produce and the environment in most countries. This has become a major food safety issue because many pesticides have not only acute toxicity, but also sublethal effects by disrupting the endocrine balance, and affecting sperm quality and reproductive development^2^,^3^,^4^,^5^,^6^,^7^. In mammalian cells, the detoxification process can be divided into several phases, including oxidation by cytochrome P450s or peroxidases (phase I), conjugation with glutathione and glucose through the action of glutathione *S*-transferases (GST) or glycosyltransferase (phase II), and extrusion of the xenobiotic conjugates and metabolites from the cell by specific membrane-associated transporters such as ABC transporters (phase III)^8^,^9^,^10^,^11^. In plants, the expression of specific genes involved in the processes related to detoxification is responsive to xenobiotic compounds such as pesticides^12^, suggesting that the intrinsic detoxification mechanisms of higher plants may be exploited to decrease pesticide residues in food produce. Brassinosteroids (BRs) are a class of phytohormones involved in the regulation of plant growth, development and stress response^13^, Previously, we found that BRs-induced stress tolerance is associated with changes in cellular redox homeostasis and expression of a wide range of stress-related genes including those encoding P450 and GST involved in metabolism of xenobiotic compounds^14^,^15^. Here, we show that BRs could enhance pesticide degradation most likely by enhancing glutathione metabolism and glutathione *S*-transferase (GST) activity via a *Respiratory burst oxidase homologue1* (*RBOH1*)-dependent pathway. Our study revealed yet another potentially important application of these plant hormones for improving the food safety of agricultural produce.

## Results

### Involvement of BR signaling in the regulation of chlorothalonil (CHT) degradation in various plants

**Figure 1:**
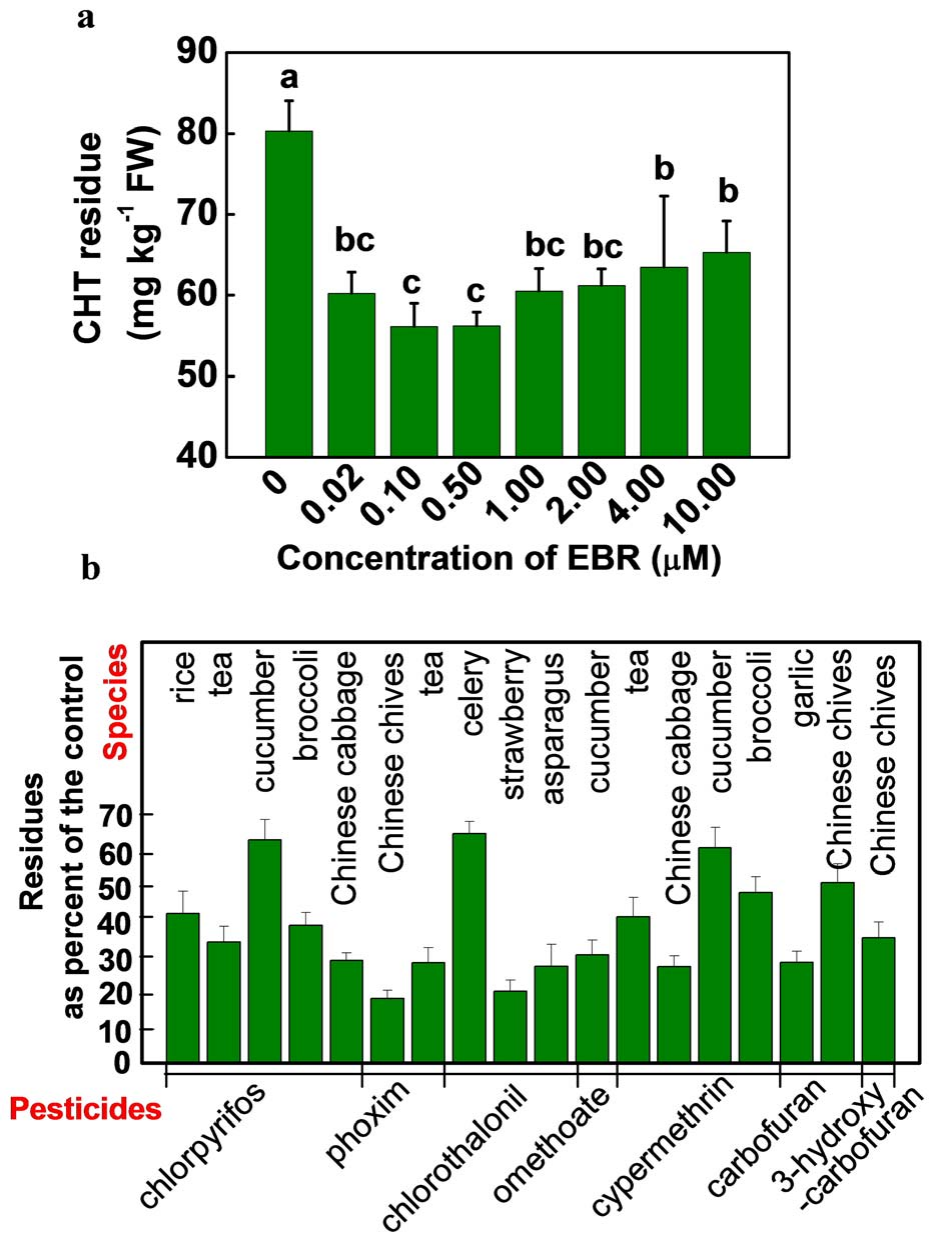
(a) Chlorothalonil (CHT) residues in tomato leaves as influenced by the application dose of 24-epibrassinolide (EBR). Plants at the 6-leaf stage were sprayed with EBR at different concentrations and then exposed to CHT at 11.2 mM. Leaves were taken at 5 d after application of CHT. (b) Effects of EBR application on the pesticide residues in various food crops. EBR at 0.1 μM was applied 6 h prior to pesticide application and pesticide residues were determined 7 d afterward. Data are means of four biological replicates (±SD).

Chlorothalonil (CHT) is among the most widely used pesticides in crop production. We first compared the effects of different concentrations of 24-epibrassinolide (EBR), an active brassinosteroid, from levels within physiological range to saturation level on CHT residues in tomato leaves. Time course of CHT residue revealed that CHT residue rarely decreased after 6 d of CHT application (Supplementary Fig. S1). EBR at the low concentration of 0.02 μM dramatically decreased the residue by 38.9% (Fig. 1a). Increasing the concentration of EBR, however, did not further accelerate CHT degradation (Fig. 1a; Supplementary Fig. S1). This result indicated that EBR concentration within physiological ranges is sufficient to upregulate the machinery of pesticide metabolism.

To determine whether BRs are general activators of pesticide metabolism in plants, we further tested with other crop plants and a variety of pesticides. EBR at 0.1 μM was co-treated with chlorpyrifos, phoxim, chlorothalonil, omethoate, cypermethrin, carbofuran or 3-hydroxycarbofuran at commercially recommended doses on rice, tea, cucumber, broccoli, asparagus, strawberry, celery, garlic, Chinese chives and Chinese cabbage plants. As found in tomato, there were significant decreases of pesticide residues in all of these crops, by an average of 34.0–71.3%, during the first 7 d after pesticide application (Fig. 1b). These results indicate that BRs are generally effective in enhancing the degradation of various pesticides in higher plants of agricultural importance.

**Figure 2:**
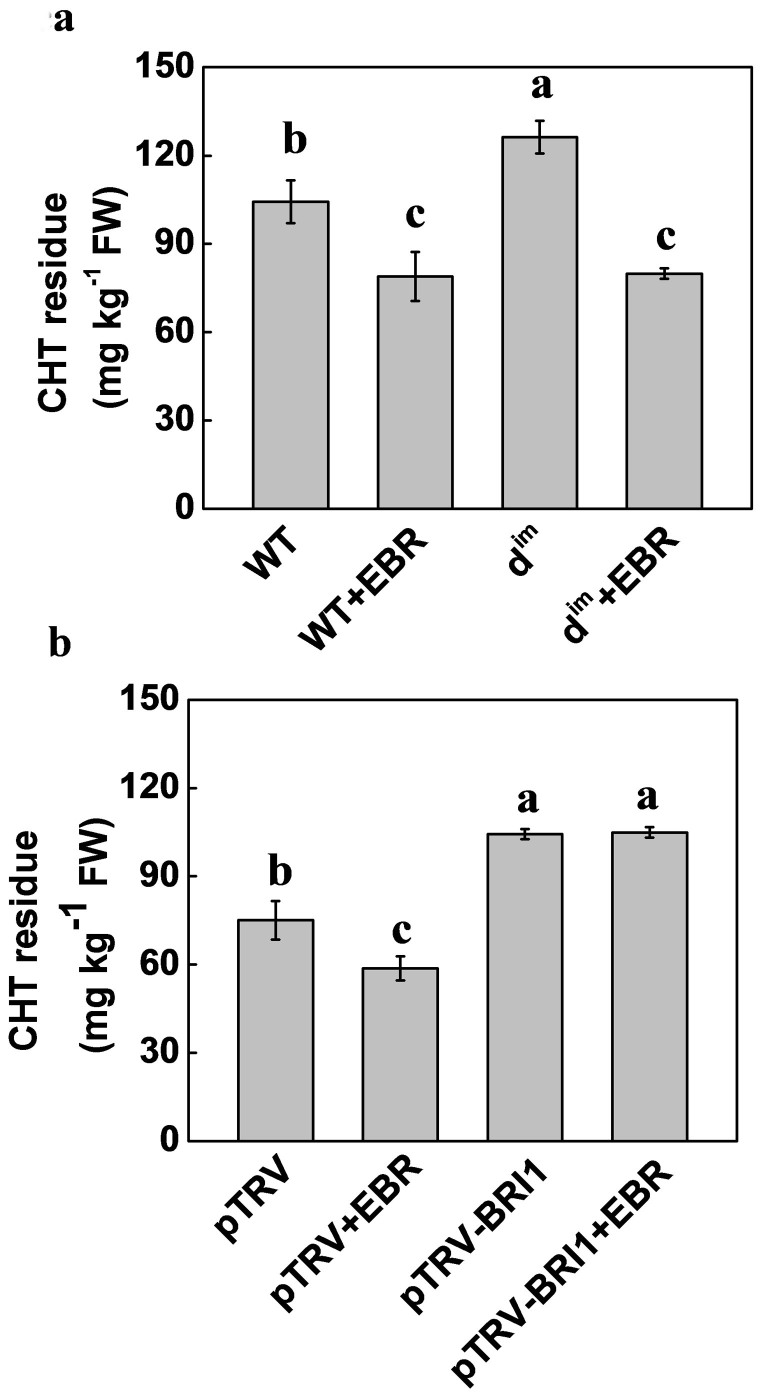
(a) Chlorothalonil (CHT) residue in the wild-type (WT) and BR-deficient mutant *d^im^* tomato plants as influenced by the application of 24-epibrassinolide (EBR). (b) CHT residues in the control (pTRV) and BRs receptor gene *BRI1*-silenced (pTRV-*BRI1*) plants as influenced by the application of EBR. EBR at 0.1 μM was applied 6 h prior to pesticide application and leaves were taken at 5 d after application of CHT at 11.2 mM. Data are means of four biological replicates (±SD). Means denoted by the same letter did not significantly differ at *P* < 0.05 according to Tukey's test.

We next examined whether endogenous BR biosynthesis and signaling affect pesticide metabolism in tomato plants by comparing between BR-deficient mutant *d^im^* and its corresponding wild-type (WT), or between the BR receptor *BRI1*-silenced (pTRV-*BRI1*) and nonsilenced (pTRV) plants for the levels of CHT residues with or without EBR application. As shown in Fig. 2a, the levels of CHT residues in the *d^im^* and the pTRV-*BRI1* plants were 21.7% and 30.2% higher than those in the WT plants and the non-silenced (pTRV) plants, respectively. Pretreatment with EBR decreased the CHT residues in the WT plants and *d^im^* plants by 31.6% and 58.1%, respectively (Fig. 2a). In contrast, silencing of *BRI1* (pTRV-*BRI1*) abolished the EBR-induced reduction in CHT residues in plants (Fig. 2b). These results strongly suggest that BRs are involved in the regulation of CHT metabolism in tomato plants.

### Plant responses to pesticide and brassinosteroids might be regulated by common signaling transduction pathway

**Figure 3:**
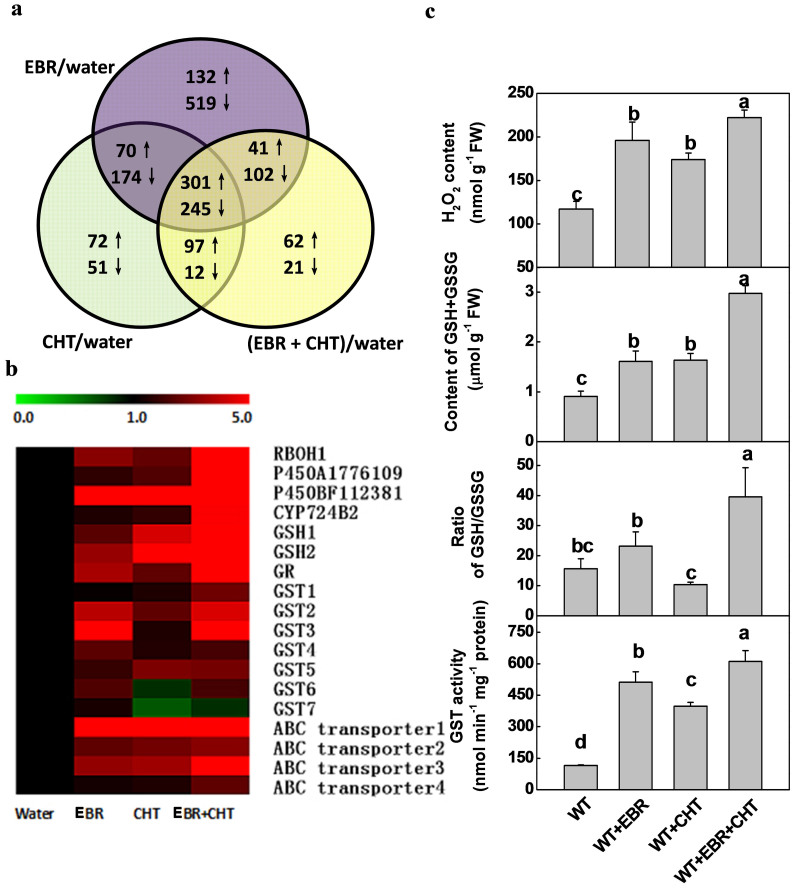
(a) Venn diagram showing the number of genes with more than 2-fold changes after treatment with EBR, CHT or EBR+CHT in comparison with the untreated controls. ↑, up-regulated; ↓, down-regulated. (b) Comparison of expression of sets of detoxification genes in EBR, CHT and EBR+CHT treated and control plants. (c) Leaf H_2_O_2_ content, glutathione homeostasis and GST activity as influenced by EBR, CHT and EBR+CHT treatments. EBR at 0.1 μM was applied 6 h prior to pesticide application and leaves were taken at 5 d after application of CHT at 11.2 mM. Data are means of four biological replicates (±SD). Means denoted by the same letter did not significantly differ at *P* < 0.05 according to Tukey's test.

The signaling pathways associated with the response to xenobiotic compounds in mammalian cells involve both xenobiotic ligand-activated transcription factors and redox sensing proteins^10^,^11^. In plants, however, no signaling components involved in plant responses to xenobiotic compounds such as pesticides have been identified. To determine the molecular responses of plant to pesticides and EBR, Affymetrix GeneChip Tomato Genome Array with more than 9,200 gene-specific probes was used to identify differentially expressed genes. A total of 1584, 1545 and 1725 genes were differentially expressed in EBR-, CHT- and EBR+CHT-treated plants, respectively. Among these differentially regulated genes, 544, 670 and 700 genes were up-regulated by EBR, CHT and EBR+CHT, respectively (Fig. 3a). Of striking interest is that 301 genes were commonly upregulated by EBR, CHT and EBR+CHT treatments and many of these genes encode proteins belonging to the catalytic category (Supplementary Table S1). In addition to those encoding cytochrome P450, GST, hydrolase and oxidoreductase, there were many upregulated genes encoding putative transcription factors or proteins associated with signal transduction, including ethylene response factor, WRKY factors, NADPH oxidase and protein kinases. Analysis of qRT-PCR further showed that both EBR and CHT differentially induced the transcripts of *RBOH1* (*Respiratory burst oxidase homologue1*, encoding NADPH oxidase), the cytochrome P450 encoding genes (accessions AI776109 and BF112381), *GSH1-2* (encoding γ-glutamyl cysteine synthetase and glutathione synthetase, respectively), *GR* (encoding glutathione reductase), *GST1-7* and *ABC1-4* (encoding ABC transporters) (Fig. 3b; Supplementary Fig. S2). Furthermore, the transcripts for most of these genes were more strongly induced by the combined EBR+CHT treatment than by the EBR or CHT alone treatment, suggesting an additive effect of BR and CHT on the induction of detoxifying mechanisms. Consistent with gene transcripts, accumulation of H_2_O_2_ and glutathione pool (reduced glutathione GSH and oxidized glutathione GSSG), and GST activity were induced by EBR and CHT (Fig. 3c; Supplementary Fig.S3). Changes of these compounds were again stronger in the EBR+CHT co-application treatment. Contrary to the EBR-induced sharp increase in GSH/GSSG ratio, there was a decrease in the GSH/GSSG ratio in CHT-exposed plants and the BR-deficient mutant *d^im^* plants (Supplementary Fig.S3). All these results suggest that BR is capable of inducing the transcripts and metabolism associated with CHT degradation in tomato plants.

### BRs enhance CHT degradation by apoplastic H_2_O_2_–dependent increases in glutathione biosynthesis and GST activity

BRs induce H_2_O_2_ production at the apoplast^15^. To examine whether BR-induced H_2_O_2_ and subsequent glutathione biosynthesis and regeneration play a role in the CHT degradation, we used diphenyleneiodonium (DPI), an inhibitor of NADPH oxidase, dimethylthiourea (DMTU), a scavenger of H_2_O_2_, and 6-aminonicotinamide (6-AN), an inhibitor of the pentose phosphate pathway to block the changes in GSH/GSSG ratio in response to EBR or CHT treatment. EBR-induced glutathione (GSH+GSSG) accumulation, GST activity and the transcripts of the genes involved were all decreased by these inhibitors or scavenger but increased by H_2_O_2_ treatment, and the effect was especially significant for GST activity (Supplementary Figs. S4 & 5). Consistent with this observation, DPI, DMTU and 6-AN all compromised EBR-induced degradation of CHT in the leaves (Supplementary Fig. 6). All these results suggested that BR-induced H_2_O_2_ accumulation and associated GSH biosynthesis and regeneration played an important role in BR-induced CHT degradation.

**Figure 4:**
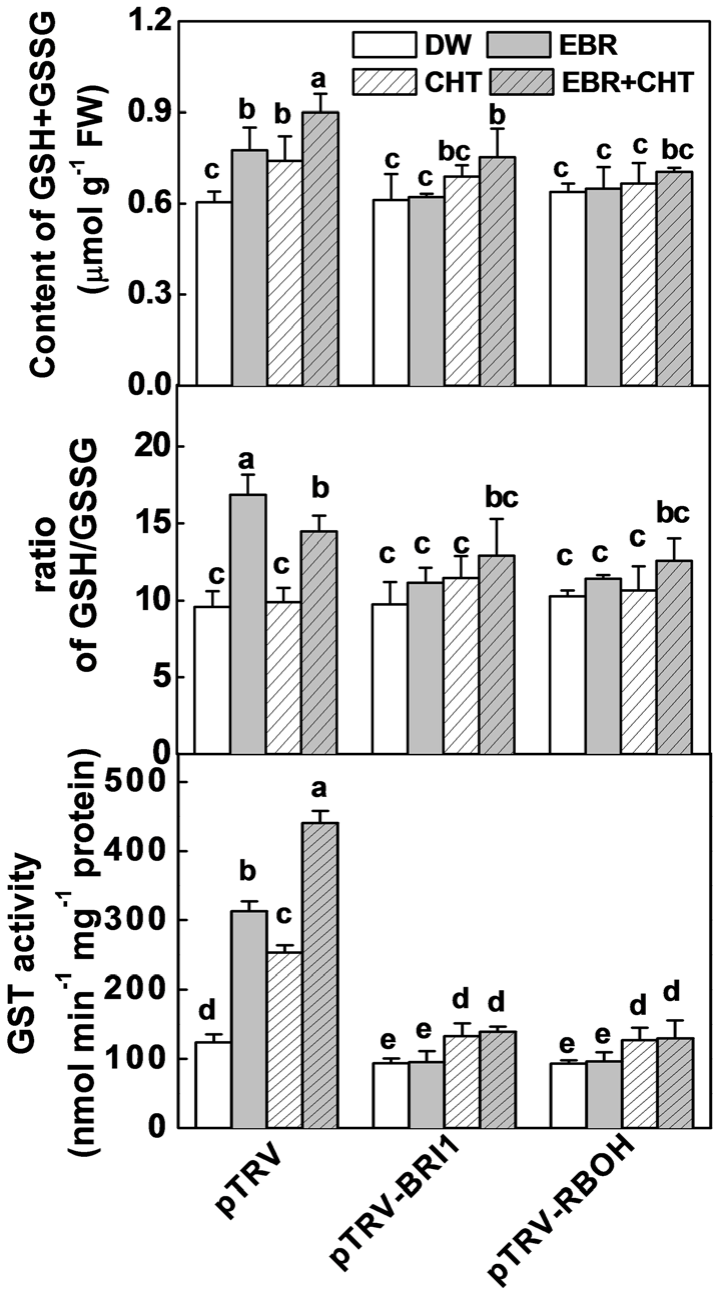
Involvement of *BRI1* and *RBOH1* in brassinosteroid (BR) and chlorothalonil (CHT)-regulated glutathione metabolism and GST activity. EBR at 0.1 μM was applied 6 h prior to pesticide application and leaves were taken at 5 d after application of CHT at 11.2 mM. Data are means of four biological replicates (±SD). Means denoted by the same letter did not significantly differ at *P* < 0.05 according to Tukey's test.

**Figure 5:**
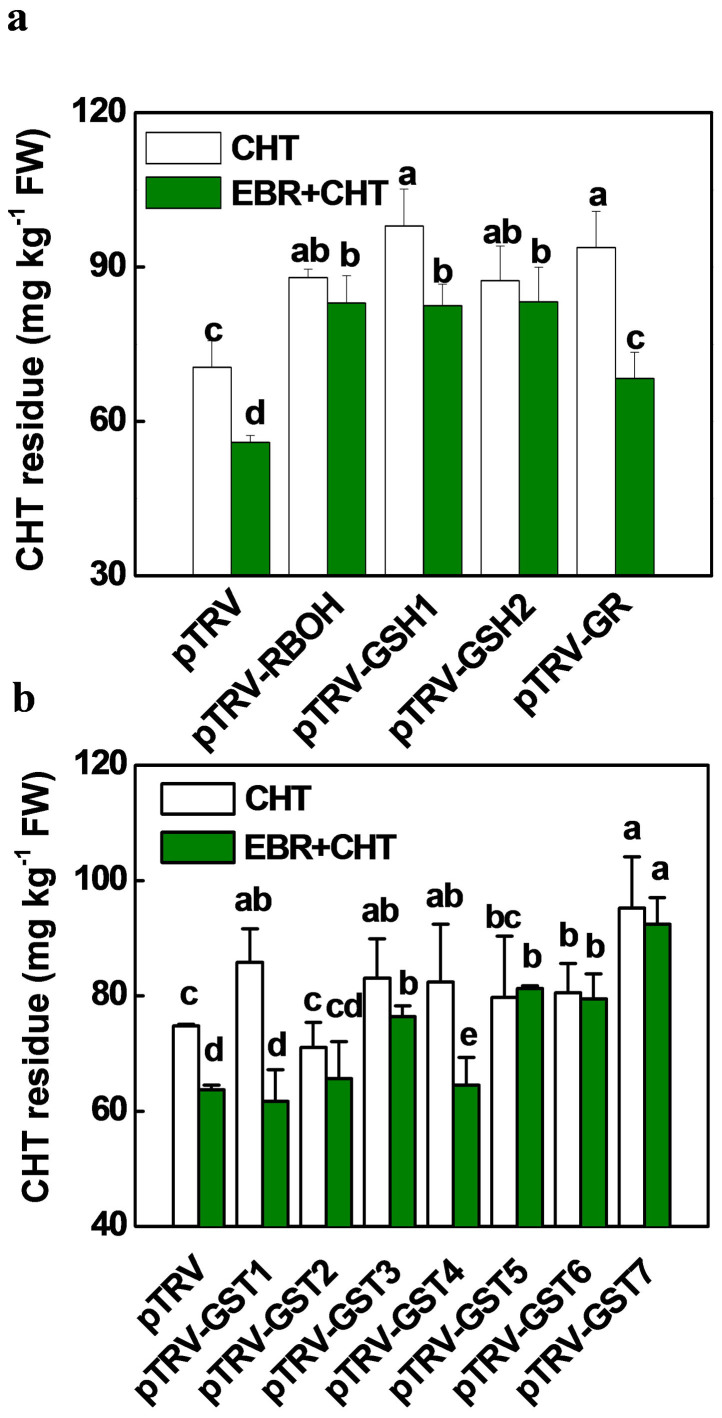
(a) Chlorothalonil (CHT) residues in *RBOH1*-, *GSH1*-, *GSH2*- and *GR*- silenced plants with or without EBR treatment. (b) Chlorothalonil (CHT) residues in *GST1-7* silenced plants with or without EBR treatment. EBR at 0.1 μM was applied 6 h prior to pesticide application and leaves were taken at 5 d after application of CHT at 11.2 mM. Data are means of four biological replicates (±SD). Means denoted by the same letter did not significantly differ at *P* < 0.05 according to Tukey's test.

To provide genetic evidence for the role of apoplastic H_2_O_2_, glutathione biosynthesis and regeneration in CHT metabolism, we silenced genes involved in the apoplastic H_2_O_2_ production (*RBOH1*), GSH biosynthesis (*GSH1* and *GSH2*) and regeneration (*GR1*) and genes encoding GSTs. Plants silenced with *BRI1* (pTRV- *BRI1*) and *RBOH1* (pTRV- *RBOH1*) all showed similar GSH and GSSG accumulation and GST activity as compared to the pTRV plants (Fig. 4). Importantly, EBR- and CHT-induced GSH and GSSG accumulation and GST activity were largely compromised in the pTRV- *RBOH1* and pTRV- *BRI1* plants (Fig. 4). Meanwhile, pTRV- *RBOH1* plants showed increased CHT accumulation as compared to pTRV plants and CHT accumulation in pTRV- *RBOH1* was not influenced by EBR application (Fig. 5a). In comparison, plants silenced for *GSH1* (pTRV-*GSH1*), *GSH2* (pTRV-*GSH2*) and *GR1* (pTRV-*GR1*) all showed increased CHT accumulation, which was associated with decreased GSH and GSSG accumulation and GST activity as compared to those of the pTRV plants (Fig. 5a, Supplementary Fig. S7). Importantly, EBR co-treatment decreased CHT residue in pTRV, pTRV-*GSH1* and TRV-*GR* plants but not in TRV-*GSH2* plants. Furthermore, EBR-induced increases in GSH and GSSG accumulation, the GSH/GSSG ratio and GST activity were abolished by the silencing of *GSH1*, *GSH2* and *GR* (Supplementary Fig. S7). All these results indicate that BR-enhanced CHT degradation is dependent not only on H_2_O_2_ production but also on biosynthesis and regeneration of glutathione.

It is known that GST conjugates glutathione to xenobiotics, thus converting the xenobiotics to nonreactive water-soluble conjugates that are easily excreted^9^. The pTRV-*GST1*, pTRV-*GST3*, pTRV-*GST4*, pTRV-*GST6* and pTRV-*GST7* plants all showed an increased CHT accumulation as compared to the pTRV plants (Fig. 5b). In contrast, CHT residues in pTRV-*GST2* and pTRV-*GST5* plants were not significantly different from those in pTRV control plants. Importantly, EBR co-treatment decreased CHT accumulation in the pTRV, pTRV-*GST1* and pTRV-*GST 4* plants, but had little effect on CHT residues in the pTRV-*GST2*, pTRV-*GST3*, pTRV-*GST6* and pTRV-*GST7* plants. A comparison of the transcripts revealed that transcript levels of *GST3, GST6* and *GST7* were affected more significantly by EBR than by CHT (Fig. 3b). Taken together, EBR-accelerated CHT degradation is also dependent on some members (e.g. *GST3, GST6* and *GST7*) of the GST gene family in tomato plants.

## Discussion

Currently, efforts to reduce health risk from pesticide residues in agricultural produce have been largely through regulations or product cancellations, and there are few studies that focused on reducing pesticide residue in intact plants, mostly due to lack of understanding of the mechanisms of pesticide metabolism and its regulation pathway in plants. As shown in this comprehensive study, altered BR levels or BR signaling significantly changed the efficiency of pesticide metabolism in plants, which was associated with altered expression of a subset of detoxifying genes, GSH biosynthesis and the GST activity (Figs. 2,3). Thus, endogenous BRs are critical for the induction of detoxifying response against pesticides.

Although many studies revealed that phytohormones are involved in the response against both biotic and abiotic stresses, little is known about their role in the regulation of xenobiotics metabolism in plants. Our previous study has shown that exogenous applied BR decreased pesticide residue in cucumber^16^. In this study, we have shown that the effects of EBR on pesticide metabolism are not unique to cucumber but also effective in a subset of crops including tomato, rice, tea and broccoli etc. with an array of pesticides (Fig. 1). Importantly, reduction of BRs level in *d^im^* mutant plants or silencing of *BRI1* (pTRV- *BRI1*), which encodes BRs receptor significantly impaired the capacity of pesticide metabolism, indicating that endogenous BRs were critical for the induction of detoxification in response to pesticides.

From microarray data, it is clear that the BR response involves induction of various detoxifying genes, especially those encoding cytochrome P450, GSH, GST and transporters, with corresponding induction of enzyme activities and this is especially significant in the presence of CHT. The similarity of transcriptional profile of CHT- and EBR- treated plants suggested that both responses shared common signaling components. In fact, an array of genes involved in hormonal, stress and redox response are regulated by both CHT and EBR. These genes included MYB-related factors, WRKY, NAC domain factor, cytochrome P450, GST and transporters and ethylene response factor. There are many reports showing the crosstalk of BR with ethylene, jasmonic and ABA^17^,^18^,^19^,^20^. Therefore, the pesticide metabolism was thought to be influenced by hormonal status, and developmental and environmental factors.

Diverse xenobiotics such as barbiturates, pesticides, herbicide safeners and chlorophenols trigger a xenobiotic defense response in plants^21^,^22^. How pesticide is perceived and the signaling is transmitted within plant cells remains elusive. In mammals, the cysteine of receptor protein for xenobiotics was modified resulting in release of *Nrf2* transcription factor into nucleus where it binds to regulatory antioxidant responsive elements, leading to the induction of genes encoding detoxifying enzymes^23^,^24^. Various xenobiotics are known to induce ROS production, and ROS can function as cellular second messengers that are likely to modulate many different genes and proteins thus leading to a variety of responses^25^. Both microarray and RT-PCR data have identified the *RBOH1* to be upregulated by CHT and BR. Consistent with the upregulation of *RBOH1*, treatment of CHT and EBR induced significant increase in H_2_O_2_. *RBOH* encoded the essential components of a plant NADPH oxidase, and is required for the ROS production at the apoplast and signal amplification in defense and stress responses^26^,^27^,^28^. We have recently found that modulation of plant stress response by BR was dependent on H_2_O_2_^15^. H_2_O_2_ plays an important role in upregulation of defense gene expression and antioxidant capacity. In accordance with this, induction of H_2_O_2_ content after treatment with CHT and EBR was associated with increased expression of *GST* and pool of GSH. More importantly, blocking the apoplastic H_2_O_2_ accumulation by the *RBOH1* silencing, NADPH oxidase inhibitor DPI or ROS scavenger DMTU all significantly inhibited the induction of *CYP724B2*, *GST2*, *GSH2* and *ABC*, the increase of GSH pool and pesticide metabolism efficiency. This suggested the involvement of apoplastic H_2_O_2_ signal in mediating the induction of detoxifying genes in response to pesticides. This hypothesis is also supported by the upregulation of several detoxifying genes, including *GST*, *P450* and *UGT* in *cat2* deficient Arabidopsis, which had increased accumulation of H_2_O_2_^29^.

H_2_O_2_ also enhances the activity of enzymes involved in sulfate assimilation and Cys or GSH biosynthesis^30^. In our study, EBR enhanced GSH biosynthesis in response to CHT treatment, probably through increasing the H_2_O_2_ signal which regulates both GSSG regeneration by GR and GSH biosynthesis^30^,^31^. Meanwhile, BR-induced H_2_O_2_ production in the apoplast could increase the CO_2_ assimilation which could enhance GSH biosynthesis by providing the carbon skeleton for sulphur assimilation^14^,^32^. The increase in the GSH level led to a higher GSH/GSSG, which may act as signal regulating the activity of transcription factors, enzymes and other proteins. It is apparent that the transcript of detoxification related gene such as *P450*, *GSH2*, *GST2* and *ABC* as well as the GST activity were all dependent on the ROS accumulation and the cellular glutathione redox statue (Supplementary Fig. S4 & 5), suggesting that the pesticide detoxification process are largely dependent on the redox homeostasis within the cells. Recently, TGA transcription factors have been implicated in a general detoxification network, presumably via activation of redox signaling^22^,^25^. The reduction of redox states induced by BR as shown by the increased GSH/GSSG ratio was correlated well with the expression of detoxifying gene expression and pesticide metabolism in this study. Therefore, pesticides like CHT may utilize a similar redox-regulated protein through thiol modification to induce detoxifying genes present in mammals.

Among the detoxification processes of pesticides, GST was the putative major player which catalyzes the conjugation of GSH to pesticides^33^. Recently, we have also shown that activity and expression of GST was preferentially induced by CHT in a concentration and time-dependent manner^34^. This indicated that pesticide response was conserved in mammals, microbes and plants. The activation sequence-1 (as-1)-like elements in the promoter of some *GST* genes has been found to be responsive to ROS^35^. In agreement with this, different *GST* genes were differentially induced by EBR and silencing of *RBOH1* or *BRI1* compromised EBR- and CHT-induced increase in GST activity. All these results provided evidence that BRs-induced changes in cellular redox statue played a critical role in the modulation of GST activity. It seems likely that different *GST* genes play different roles in the metabolism of different pesticides or subject to the regulation of BRs or redox homeostasis differentially as evidenced by the different induction of GST gene transcript and the CHT residue results in the GST-VIGS plants (Fig. 5).

Lethal and sublethal poisoning due to pesticide residues on crop produce is a serious threat to human health - a problem that is especially widespread in the developing countries due to poor regulations and use of older generations of pesticides. This challenge has remained largely unresolved to date. Here, we have demonstrated that BRs are capable of mitigating residues of a variety of pesticides in different crops in addition to their role in plant growth, development and stress tolerance^13^. In-depth experiments on tomato further revealed that BRs enhanced pesticide degradation most likely by enhancing glutathione metabolism and GST activity via a *RBOH1*-dependent pathway (Fig. 4). This function of BRs appears to be applicable for a variety of pesticides in a wide range of crops. This discovery may provide us with the possibility of minimizing pesticide residues on crop produce and the associated risks. To our knowledge, this is the first report with genetic evidence showing that pesticide degradation by an enzymatic system in higher plants is regulated by the phytohormone signaling. In addition to the foliar application of BRs, other means such as genetic engineering of BR biosynthesis and signaling could be exploited to minimize the risk of pesticide contamination in food crops. Recently, BRs were recognized and explored as a biotechnological target for enhancing crop yields and stress tolerance^36^ and our findings probably offer another exciting potential for improving the safety of human foods.

## Methods

### Plant materials and virus-induced gene silencing (VIGS) of detoxification related genes

Tomato seeds (*Solanum lycopersicum* cv Condine Red) and its corresponding BR-deficient mutant (*d^im^*) were obtained from TGRC (Tomato Genetic Resource Center) at University of California, Davis. The plants were raised in plastic pots (15 cm diameter and 15 cm height, one seedling per pot) filled with a mixture of peat and vermiculite (7:3, v/v) and were watered daily with Hoagland nutrient solution. The growth conditions were as follows: a 12-h photoperiod, temperature of 25/20°C (day/night), and photosynthetic photon flux density (PPFD) of 600 μmol m^−2^ s^−1^.

The tobacco rattle virus (TRV) VIGS vectors used for silencing of tomato genes were constructed as described previously^37^. The cDNA fragments for *BRI1, RBOH1*, *GSH1, GSH2, GR* and *GST1-7* were PCR-amplified using the gene-specific primers listed in Supplemental Table 2. The amplified fragment was digested with SacI and XhoI and ligated into the same sites of pTRV2. The resulting plasmid was mobilized into *Agrobacterium tumefaciens* GV310. VIGS was performed by infiltration on 15-d-old seedlings with a mix of pTRV1- and pTRV2-carrying *Agrobacterium tumefaciens*. Plants were then kept at 23/21°C under PPFD of 200 μmol m^−2^ s^−1^ for 30 d before they were used for the experiments.

### Chemical treatments

When the 6^th^ true leaf fully expanded, the plants were pretreated with different concentrations of 24-epibrassinolide (EBR, Sigma, USA). After 6 h, the plants were treated by application of chlorothalonil (CHT) at 11.2 mM with 30 mL per plant. Distilled water was sprayed onto tomato leaves as a control treatment. To determine the role of H_2_O_2_ and glutathione homeostasis in the regulation of pesticide metabolism, we pretreated leaves with 100 μM diphenyl iodonium (DPI)^38^, 5 mM dimethylthiourea (DMTU), and 5 mM 6-aminonicotinamide (6-AN)^39^. Then 0.1 μM EBR and CHT were applied 8 h and CHT 14 h later, respectively. Plant samples were harvested 5 d after the CHT treatment for analysis of CHT residues. The plants were also sampled at 5 d after pesticide treatment for molecular and biochemical analysis.

To investigate the effects of EBR on the degradation of various organophosphorus, organochlorine and carbamate pesticides on various crops, plants of rice, tea, broccoli, strawberry, celery, Chinese chives, asparagus, cucumber, garlic and Chinese cabbage grown in greenhouse were first sprayed with distilled water or 0.1 μM EBR and 6 h later, with chlorpyrifos, phoxim, chlorothalonil, omethoate, cypermethrin, carbofuran and 3-hydroxycarbofuran at commercially recommended doses. After 7 d, tissue samples were taken for the analysis of pesticide residues.

### Pesticide residue analysis

To determine pesticide residues in the tested plants, 25 g (fresh weight) of tissue were homogenized for 2 min in 50 mL acetonitrile. The liquid phase was transferred into a separatory funnel containing 5–7 g of anhydrous sodium sulfate and mixed for 5 min. The acetonitrile extract was collected and concentrated to near dryness, and then dissolved in 5 mL acetone. Chlorpyrifos, phoxim, omethoate, chlorothalonil, and cypermethrin were quantitatively measured on an Agilent 6890 N gas chromatography system (Agilent Technologies, Palo Alto, CA, USA), equipped with FPD or ECD and a DB-17 silica capillary column. For the determination of carbofuran and 3-hydroxycarbofuran, the concentrated mixture was transferred to a Supelclean LC-NH2 SPE (Supelco, Poole, UK), eluted with 4 mL of methanol-methylene dichloride (1:99, v/v), concentrated to near dryness and then dissolved in methanol for analysis on an Agilent 1200 high pressure liquid chromatography (Santa Clara, CA, USA) with FLD equipped with a ZORBAX SB-C18 column (25 cm × 4.6 mm × 5 mm) after derivatization with *O*-phthaladehyde.

### Biochemical analysis

H_2_O_2_ was extracted from leaf tissues with 0.2 M HClO_4_. The homogenate was centrifuged at 2,700 *g* for 30 min at 4°C and the supernatant was collected, adjusted to pH 6.0 with 4 M KOH and centrifuged at 11,000 *g* for 10 min at 4°C. The supernatant was placed onto an AG1x8 prepacked column (Bio-Rad, Hercules, CA) and H_2_O_2_ was eluted with 4 mL double-distilled H_2_O. The sample (800 μL) was mixed with 400 μL reaction buffer containing 4 mM 2,2′-azino-di(3-ethylbenzthiazoline-6-sulfonic acid) and 100 mM potassium acetate at pH 4.4, 400 μL deionized water, and 0.25 U of horseradish peroxidase. The content of H_2_O_2_ was measured at OD_412_ as described previously^40^.

Reduced and oxidized glutathione (GSH and GSSG) were determined as described previously. Briefly, plant leaf tissue (0.2 g) was homogenized in 2 mL of 2% metaphosphoric acid containing 2 mM EDTA and the homogenate was centrifuged at 4°C for 10 min at 14,000 *g*. After neutralization with 0.5 M phosphate buffer (7.5), oxidized glutathione (GSSG) and reduced glutathione (GSH) in the supernatant were quantified using an enzymatic recycling assay as described in Queval & Noctor^41^.

### Enzyme activity assay

Glutathione reductase (GR) and glutathione *S*-transferase (GST) were extracted from 0.3 g of leaves with 2 mL extraction buffer (50 mM, potassium phosphate, pH 7.5) containing 10 mM KCl, 1 mM EDTA, 5 mM dithiothreitol (DTT), 0.5 mM AEBSF and 1:4 (w/w) polyvinypolypyrrolidone (insoluble PVPP). The activity of GR was measured according to Foyer & Halliwell^42^ based on the rate of decrease in the absorbance of NADPH at 340 nm. The activity of GST was assayed spectrophotometrically according to Habig & Jakoby^43^. Spectrophotometric analysis was conducted on a SHIMADZU UV-2410PC spectrophotometer (Shimadzu, Kyoto, Japan).

### RNA extraction and qRT-PCR for gene expression analysis

Total RNA was isolated from tomato leaves using TRIZOL reagent (Sangon, China), and the cDNA template for qRT-PCR was synthesized using a RevertAid™ first strand cDNA synthesis kit (Fermentas) by following the manufacturer's instructions. qRT-PCR was performed with an iCycler iQ Multicolor Real-time PCR detection system (Bio-Rad, Hercules, CA). Gene-specific primer pairs were designed with Primer Premier 5.0 (Premier Biosoft International, Palo Alto, CA) and are listed in Supplemental Table 3. Tomato *actin* gene was used as an internal control. Relative gene expression was calculated according to Livak & Schmittgen^44^.

### Microarray analysis

Affymetrix GeneChip Tomato Genome Array, which is designed specifically to monitor gene expression in tomato, was used for analyzing the gene expression at the whole genome level (http://www.affymetrix.com/products services/arrays/specific/tomato.affx). RNA was extracted from leaves treated with distilled water, EBR, CHT or EBR+CHT using a Qiagen RNeasy kit according to the manufacturer's instructions. Each treatment had three biological replicates. cDNA was prepared using the two cycle target labeling procedure and was used for further synthesis of labeled target copy RNA by in vitro transcription as described in the Affymetrix GeneChip manual. All procedures for probe preparation, hybridization, washing, staining, and scanning of the GeneChip Tomato arrays, as well as data collection, were performed at the Beijing Biochip National Engineering Research Center, Beijing, China. Signal summarization, normalization, and background subtraction were performed using Robust Multichip Analysis^45^ in the affymetrix package with default parameters. The statistical test for differentially expressed genes was performed using the LIMMA (linear models for microarray) package^46^. Genes showing a 2-fold change in the expression level between treatment and control at *P* < 0.05 were defined as significantly differentially expressed genes. Gene Ontology analysis was carried out by using the BLAST tool in order to match the tomato transcripts to the closest homologs of Arabidopsis with an e value of less than e^−5^.

### Statistical analysis

Data were subjected to analysis of variance, and the means were compared using Tukey's test at the 5% level.

## Author Contributions

Z.C., J.G. and J.Y. designed the research; Y.Z., X.X., G.Y., J.W., J.W., M.W., Y.Y., K.S. and Y.Y. performed the experiments; Z.C., J.G. and J.Y. wrote the manuscript.

## Supplementary Material

Supplementary Information Supplemental tables and figures
