# Supplementary material for: Brassinosteroids play a critical role in the regulation of pesticide metabolism in crop plants
Source: Sci Rep. 2015 Mar 12;5:9018. doi: 10.1038/srep09018 (PMC4356967; doi:10.1038/srep09018)

Title:

Brassinosteroids play a critical role in the regulation of pesticide metabolism in crop plants

Authors:

Yanhong Zhou[1,5], Xiajian Xia[1,5], Gaobo Yu[1,5], Jitao Wang [1], Jingxue Wu [1], Mengmeng Wang [1], Youxin Yang [1], Kai Shi [1], Yunlong Yu[2], Zhixiang Chen[3] , Jay Gan [4], Jingquan Yu [1,*]

[1]*Department of Horticulture, Zijingang Campus, Zhejiang University, 866 Yuhangtang Road, Hangzhou, 310058, P.R. China*

[2]*Institute of Pesticide & Environmental Toxicology, Zijingang Campus, Zhejiang University, 866 Yuhangtang Road, Hangzhou, 310058, P.R. China*

[3]*Department of Botany & Plant Pathology, Purdue University, West Lafayette, IN 47907-2054, USA*

[4]*Department of Environmental Science, University of California Riverside, Riverside, CA 92521, USA*

[5]*These authors contributed equally to this work.*

* *To whom correspondence should be addressed. E-mail: jqyu@zju.edu.cn*

Table S1. Up-regulated genes selected in EBR, CHT or EBR&CHT treatment

GeneBank Il	Gene description	Fold change		
Cytochrome P450		**EBR**	**CHT**	**EBR+CHT**
AI776109	cytochrome-c oxidase activity	1.7415	2.2907	5.1313
BF112381	cytochrome-c oxidase activity	5.5507	5.6309	8.0496
Oxidoreductase				
AF088276.1	NADPH oxidase activity	2.5578	2.6485	5.8656
AY008278.1	lipoxygenase	2.3882	2.6438	3.3803
BG627719	oxidoreductase activity	3.855	2.8073	4.1324
AF146691.1	Eli3 protein	3.0996	2.9781	4.1405
CK715617	oxidoreductase activity	6.8168	4.7235	6.4869
CN384480	peroxidase	5.5564	5.3219	6.6151
BM410158	oxidoreductase activity, sterol carrier activity	1.8222	2.0917	2.6426
BG627812	3-oxo-5-alpha-steroid 4-dehydrogenase activity	3.3503	4.1613	4.716
AI776010	arsenate reductase (glutaredoxin) activity	2.2675	2.7736	3.3164
Hydrolase				
AF020390.2	catalytic activity, hydrolase activity	2.6215	3.5347	4.596
AW093105	hydrolase activity, ATP binding	30.9665	20.6263	50.4062
X79337.1	hydrolase activity, endonuclease activity	32.9911	34.0407	42.463
X79338.1	hydrolase activity, nuclease activity	2.0745	2.3981	2.8656
CK468696	chitinase activity	6.2805	4.1118	7.6126
Transferase Activity				
CN385367	thiol-disulfide exchange intermediate activity	4.788	4.3973	5.645
AW036032	glutathione transferase activity	2.6914	2.6734	2.935
CK468710	glutathione transferase activity	5.1388	3.4619	4.8882
BG627684	glutathione transferase activity	9.6957	3.7924	5.9522
AY081905.1	N-hydroxycinnamoyl transferase THT1-3	7.7508	4.7491	9.1679
AY007559.1	transferase activity	14.9118	8.1856	11.6772
AW036070	N-acetyltransferase activity	2.7197	2.846	3.2293
BI205190	UDP-glycosyltransferase activity	6.0548	3.0434	5.1568

Transporter Activity				
AY026343.1	oxygen transporter activity	6.6267	4.0534	6.0643
AY731066.1	transporter activity	11.5998	5.9842	10.1278
AI781372	protein transporter activity, conjugating enzyme	1.6964	2.4052	2.8667
CK720579	purine transporter activity	3.0676	2.8781	3.6348
Ion Binding				
AI775413	calcium- and calmodulin-dependent protein kinase activity	3.0676	2.0447	2.2871
AY642285.1	regulator of gene silencing	13.8157	5.0619	11.9526
X55193.1	9612 protein, calcium ion binding	7.3558	16.7857	26.8781
Z68185.1	metallothionein-like protein	2.9688	4.4508	4.8496
BI203707	copper ion binding	1.6886	4.9358	6.0638
Transcription Factor				
AJ277944.1	SANT/MYB domain protein	5.0883	2.0452	3.4043
X99210.1	myb-related transcription factor	4.2032	2.5987	3.7005
AY044236.1	ethylene-responsive factor 1	2.0414	2.1668	2.4306
AJ715788.1	anaerobic basic leucine zipper protein	3.567	2.4517	3.8084
AY157063.1	WRKY transcription factor IId-4	2.8939	2.8722	3.4872
AY077626.1	transcription factor activity	2.0031	4.2356	5.1793
BM413526	ethylene response factor 1	2.1864	2.2521	2.5841
Signal Transducer Activity				
CK715617	protein serine/threonine kinase activity	6.8168	4.7235	6.4869
J04099.1	proteinase inhibitor I	1.7282	4.8397	5.1086
X94946.1	proteinase inhibitor II	2.1967	7.481	9.158
BG628357	signal transducer activity	4.4591	3.1082	4.1492
AF083253.1	cysteine protease inhibitor	0.9753	4.7288	6.0271

Data are means of four biological replicates

Table S2. Primers used for virus induced gene silencing

Gene	Primer pairs
BRI1	F: CGCGGATCCACCCAACTCTGCTTCAGAAC
	R: CGGGGTACCAGAACAAACACCAAAGATAGAG
RBOH1	F: AAGAATGGGGTTGATATTGT
	R: CTCTGACTTATTCCTTAC
GSH1	F: TGCTCTAGAGAACCCTGCGACCCAT
	R: CGCGGATCCAAGACCAGCACGGAAC
GSH2	F: TGCTCTAGACCAGCCAAACGTAAAG
	R: CGCGGATCCAAGTCCACGAAGAGGG
GR	F: TGCTCTAGAAGCCATAGAGGTTGACGA
	R: CGCGGATCCTCTGCTGCTGTAGGGTGA
GST1	F: TGCTCTAGAGTGAGTTCGTCGGAGTTA
	R: CGCGGATCCAGTTTGAGTGATGCCAGT
GST2	F: CGAGCTCTGGGCACAAAAGAAAGTA
	R: CCGCTCGAGTAATCCCTCTACCACCGA
GST3	F: TGCTCTAGAGAACAAGAGGCAGGTA
	R: CGCGGATCCCAAGATAAAGGCAGAAT
GST4	F: TGCTCTAGAATTGTTGAGGAGGTTGTG
	R: CGCGGATCCTGCGGTATGCCCTTATTT
GST5	F: CCGGAATTCCTACTACACGACGACAA
	R: CCGCTCGAGGGCACTGAGCCAAAGA
GST6	F: TGCTCTAGATTGGGCTGATTACATTG
	R: CGCGGATCCTTCTGCCTTAGCACTTT
GST7	F: TGCTCTAGAGGATTGCCCTAGCTGAA
	R: CGCGGATCCAGTATGGTTTGTCTCCC

F indicates forward and R indicates reverse.

Table S3. Primers used for real time RT-PCR assays

Gene	Primer pairs
Actin	F:TGGTCGGAATGGGACAGAAG
	R:CTCAGTCAGGAGAACAGGGT
GST1	F: CTGCATTCTGGGTGGGT
	R: CTCGGCTACTTCGTTCA
GST2	F: ACATTGCCAAACTACGG
	R: ACCTCTGACATCCTCCC
GST3	F: ATGGACAACAAAGGGAGGAG
	R: CCAATCAACGCAATATCCAC
GSH1	F: CTGCATTCTGGGTGGGT
	R: CTCGGCTACTTCGTTCA
GSH2	F: TTGATGCGAACAAAGGTCTC
	R: ATAGTGTCAACGCGCAAGAC
RBOH1	F:CATTTGATTTGGGACA
	R:CTTCAACAAACTCCTCC
ABC1	F: TCATTGAGGAGGTCATGGAA
	R: AATCCAGAGGTTGGCTCATC
ABC2	F: AGGTTGACGATTGCAGTTGA
	R: TCTCATCACAATTGCAGCAG
ABC3	F: GCACTTGTGCAGGAGTTTGT
	R: TGTCAGCCGTTTACGTTGTT
ABC4	F: TCATTGAGGAGGTCATGGAA
	R: AATCCAGAGGTTGGCTCATC
CYP724B2	F: TCGCATAAGGGTGAGT
	R: CCTGAGTGGCAAGACA
P450AI77610	F:TGGATTGCACATGCCACATT
	R: TTGCGATAGGTATTTCCTTGAC
P450BF11238	F:CACATGCTGCATACAAGGGTC
	R: CTGGTTGTTCCAATGGTGCT

F indicates forward and R indicates reverse.

Figure legends

Fig. S1. Time-course of chlorothalonil (CHT) residues in tomato leaves as influenced by the application dose of 24-epibrassinolide (EBR). Plants at the 6-leaf stage were sprayed with EBR at different concentrations and then exposed to CHT at 11.2 mM. EBR at 0.1 μM was applied 6 h prior to CHT application and CHT residues were determined 7 d afterward. Data are means of four biological replicates (±SD).

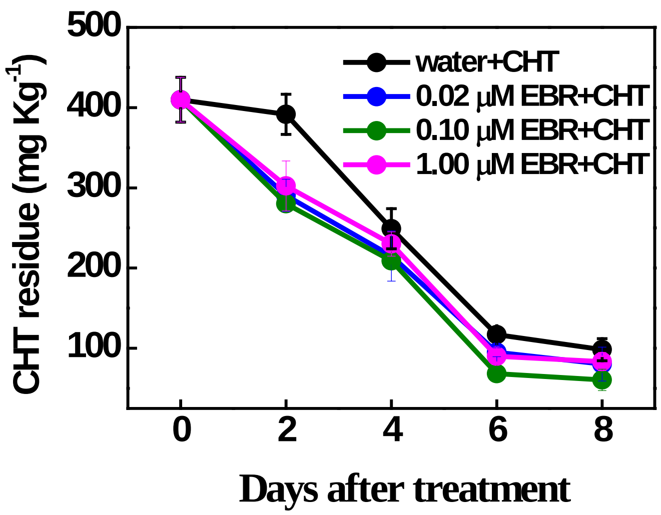

Fig. S2. Transcripts of detoxifying genes as influenced by 24-epibrassinolide (EBR), chlorothalonil (CHT) and EBR+CHT treatments in wild-type (WT) and BR-deficient mutant *d*^{im} plants. EBR at 0.1 μM was applied 6 h prior to pesticide application and leaves were taken at 5 d after application of CHT at 11.2 mM. Data are means of four biological replicates (±SD). Means denoted by the same letter did not significantly differ at *P* < 0.05 according to Tukey's test.

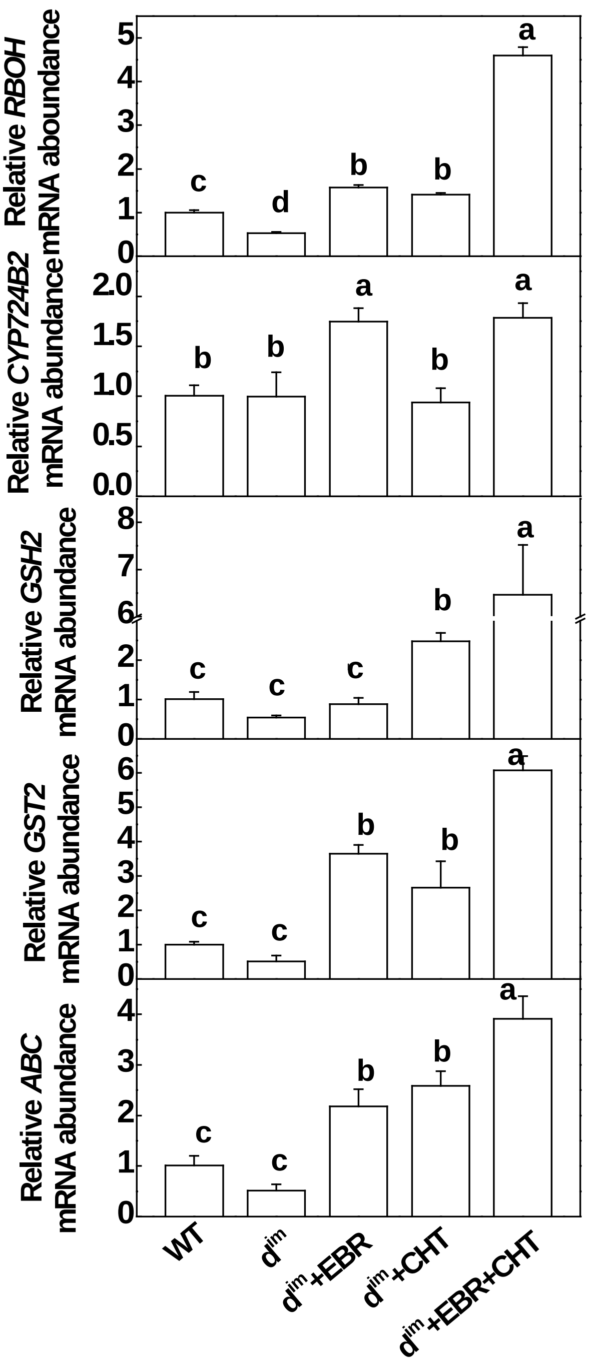

Fig. S3. H_2O_2, glutathione homeostasis and GST activity as influenced by 24-epibrassinolide (EBR), chlorothalonil (CHT) and EBR+CHT treatments in wild-type (WT) and BR-deficient mutant d^{im} plants. EBR at 0.1 μM was applied 6 h prior to pesticide application and leaves were taken at 5 d after application of CHT at 11.2 mM. Data are means of four biological replicates (±SD). Means denoted by the same letter did not significantly differ at $P < 0.05$ according to Tukey's test.

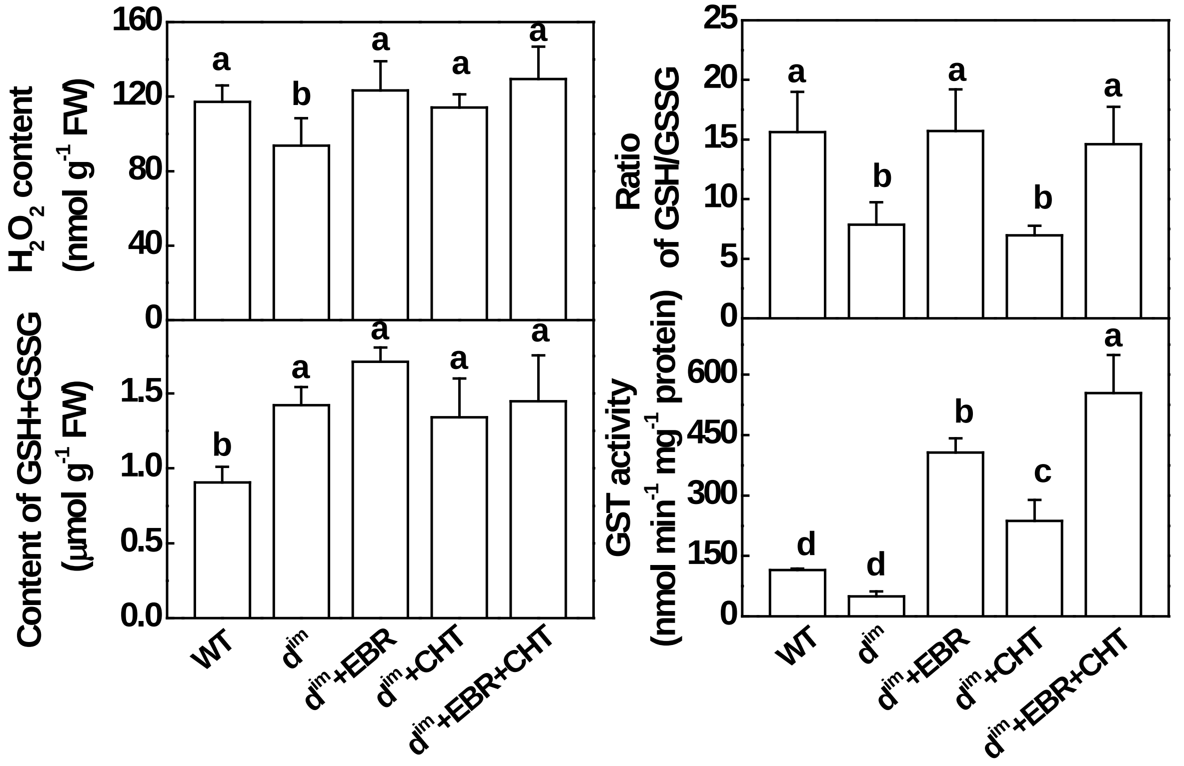

Fig. S4. H_2O_2 accumulation and transcripts of detoxifying genes as influenced by H_2O_2 and glutathione homeostasis in tomato leaves. Plant were treated with distilled water (DW), EBR or H_2O_2 with or without pretreatment of inhibitor of NADPH oxidase (DPI), scavenger of H_2O_2 (DMTU) or blocker of glutathione homeostasis (6-AN) and then applied with chlorothalonil (CHT). EBR at 0.1 μM was applied 6 h prior to pesticide application and leaves were taken at 5 d after application of CHT at 11.2 mM. Data are means of four biological replicates (±SD). Means denoted by the same letter did not significantly differ at $P < 0.05$ according to Tukey's test.

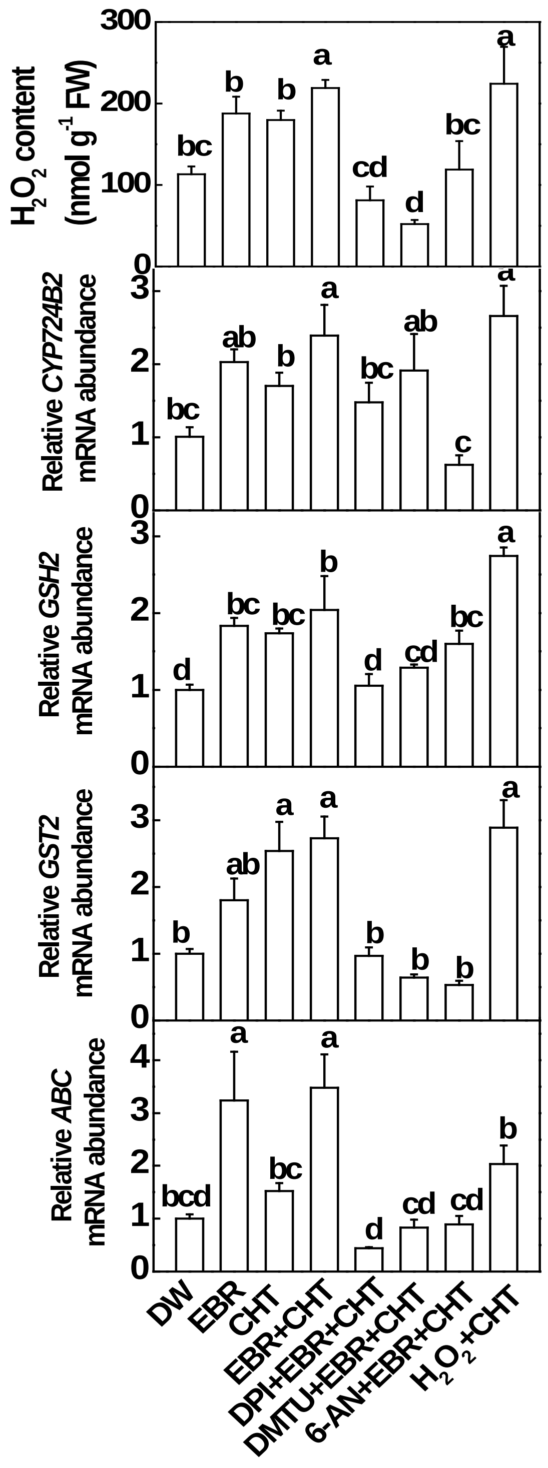

Fig. S5. Glutathione accumulation, GR and GST activity as influenced by H_2O_2 and glutathione homeostasis in tomato leaves. Plant were treated with EBR or H_2O_2 with or without pretreatment of inhibitor of NADPH oxidase (DPI), scavenger of H_2O_2 (DMTU) or blocker of glutathione homeostasis (6-AN) and then applied with chlorothalonil (CHT). EBR at 0.1 μM was applied 6 h prior to pesticide application and leaves were taken at 5 d after application of CHT at 11.2 mM. Data are means of four biological replicates (±SD). Means denoted by the same letter did not significantly differ at P < 0.05 according to Tukey's test.

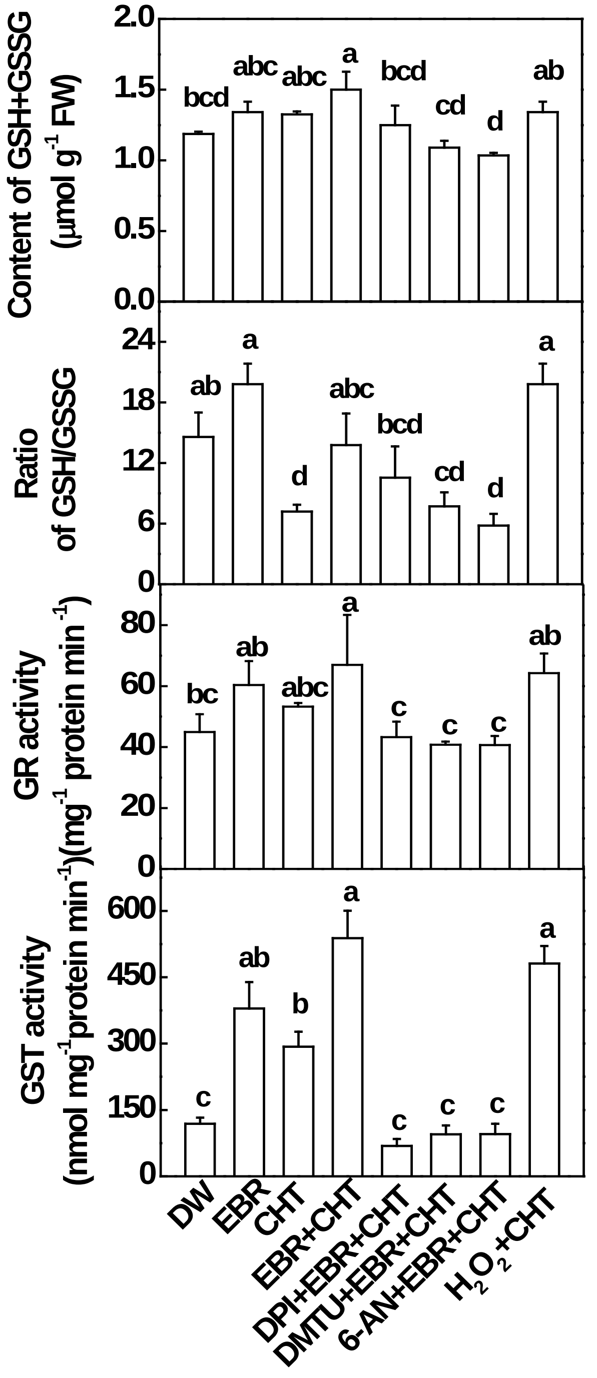

Fig. S6. Chlorothalonil (CHT) residues as influenced by H_2O_2 and glutathione homeostasis in tomato leaves. Plant were treated with EBR or H_2O_2 with or without pretreatment of inhibitor of NADPH oxidase (DPI), scavenger of H_2O_2 (DMTU) or blocker of glutathione homeostasis (6-AN) and then applied with CHT. EBR at 0.1 μM was applied 6 h prior to pesticide application and leaves were taken at 5 d after application of CHT at 11.2 mM. Data are means of four biological replicates (±SD). Means denoted by the same letter did not significantly differ at $P < 0.05$ according to Tukey's test.

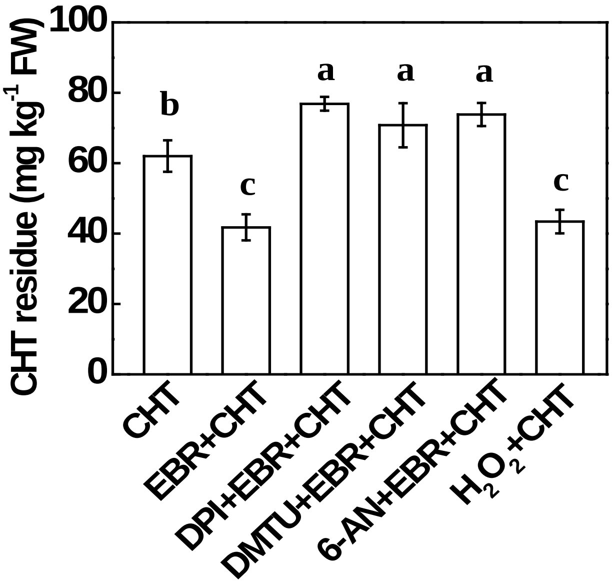

Fig. S7. GSH homeostasis and GST activity as influenced by the silencing of *GSH1*, *GSH2* and *GR* as well as the EBR treatment. EBR at 0.1 μM was applied 6 h prior to pesticide application and leaves were taken at 5 d after application of CHT at 11.2 mM. Data are means of four biological replicates (±SD). Means denoted by the same letter did not significantly differ at $P < 0.05$ according to Tukey's test.

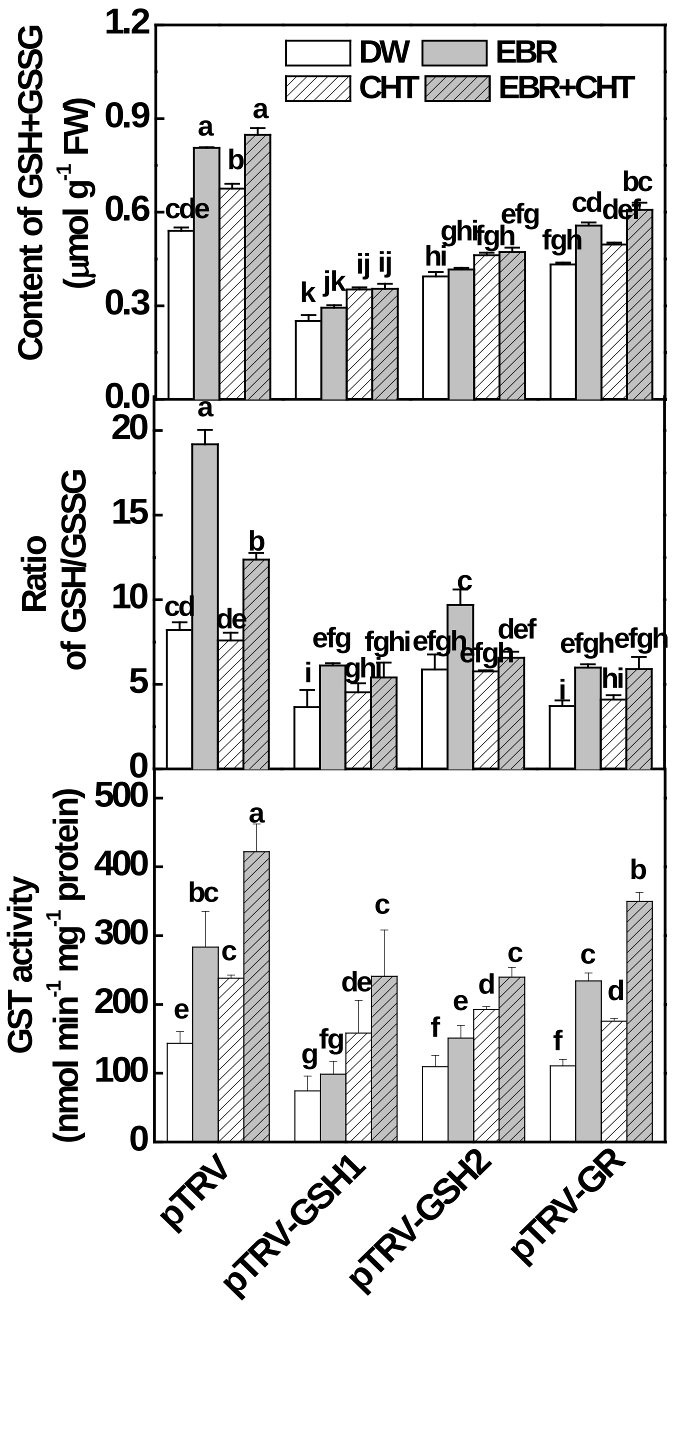

Supplement: Supplementary Information — Supplemental tables and figures [file srep09018-s1.pdf]
